# Significant Others, Knowledge, and Belief on Smoking as Factors Associated with Tobacco Use in Italian Adolescents

**DOI:** 10.1155/2013/968505

**Published:** 2012-11-27

**Authors:** Fiammetta Cosci, Vincenzo Zagà, Giuly Bertoli, Aquilele Campiotti

**Affiliations:** ^1^Department of Psychology, University of Florence, Via di San Salvi 12, 50135 Florence, Italy; ^2^Department of Pneumatology, AUSL of Bologna, Via Tiarini 10, 40129 Bologna, Italy; ^3^Service of Health Promotion, ASL of Milano 1, Via al Donatore di Sangue 50, Magenta, 20013 Milan, Italy

## Abstract

Tobacco use is dramatically increasing among youth. Growing attention has been addressed towards possible predictors of smoking in such a population. We evaluated a sample of Italian adolescents to verify whether adults and peers might influence their smoking status. Cross-sectional study was conducted in 16 schools of North Italy. Data were collected from 2001 to 2010 by means of a self-administered questionnaire on sociodemographic data and individual/social possible predictors of smoking. 2,444 students (56.7% boys; 43.3% girls; mean = 14.32 ± 1.384 years) were analysed. 607 (24.8%) were current smokers; 1,837 (75.2%) were nonsmokers. The presence of smokers in the family, seeing teachers who smoke, the influence of friends, and the feeling of inferiority were predictors of youth smoking as well as unawareness of nicotine dangerous action to health. Running the logistic multivariate analysis with all the variables listed above in the same model, the strongest predictors of smoking were as follows: being unaware that pipe/cigar is harmful to health as cigarettes; not knowing that passive smoking is harmful to the growth of children; having seen teachers smoking. The present findings help to identify the variables that might favour smoking in youth. Such variables should become the target of prevention programs.

## 1. Introduction

Tobacco use is one of the major preventable causes of death in the world. The World Health Organization attributes over four million deaths a year to tobacco and this figure is expected to rise to 10 million by 2030. In the developed countries, tobacco use is dramatically increasing among youth; the phenomenon has been described as a “paediatric disease” and a “paediatric epidemic”. Nearly 25% of students aged 13–15 years smoke and have smoked their first cigarette before the age of 10 [1]. If this pattern continues, tobacco use will result in the deaths of 250 million children and young people alive today. Moreover, cigarette smoking has a high morbidity in young people causing upper respiratory tract infections, reduced lung growth, and retardation in the level of maximum lung function. Of particular concern is also the association with health risk behaviours, including high-risk sexual behaviour and substances use [2]. Finally, individuals who begin smoking at a young age are more likely to develop high nicotine dependence than those who start later [3]; this would indicate a greater chance of smoking through adulthood [4].

In this framework, sophisticated programs of youth behaviour surveillance, including tobacco use, have been implemented and increasing attention has been raised towards possible predictors of tobacco smoking, such as individual, social, and societal factors. 

Individual predictors favouring tobacco smoking include: demographic variables, for instance, older age [5], male gender [6], white race [7]; psychological symptoms, such as depression [7], anxiety, conduct disorders, substance abuse [8]; inadequate health-related behaviour [9]; attitudes like low psychological sense of well-being or life satisfaction [7]; personality traits as risk taking and rebelliousness [8]; the lack of knowledge of smoking effects [9]; having smoking-related positive beliefs about the benefits of smoking [10]. Moreover, intentions to start and quit smoking and school-related variables [9] such as having poor academic performance [5] and attending public rather than private schools [11] seem to favour the smoking onset. 

Social factors inducing tobacco consumption include smoking behaviour of parents, siblings, peers, and significant adults [9, 12]. Recently, Mak and colleagues [13] found that parental smoking and having a smoking best friend were associated with adolescent current smoking, ever smoking, and intention to initiate smoking. Having a smoking best friend was also associated with reinitiating and quitting smoking. However, although the link between peers/significant adults and adolescent smoking is widely accepted, it is not yet completely clear which variable has the strongest influence [5, 8, 11]. Still in the frame of significant adults, smoking by teachers appears to be a predictor for students smoking [14]. In particular, the exposure to teachers' smoking outside the school seems to have a greater effect than inside it [15].

Also family characteristics, social support, and socioeconomic status seem to exert an important role [9]. For instance, adolescents who perceive that both parents would respond negatively and be upset by their smoking are less likely to smoke [16]; an authoritative parenting style seems associated with children's less frequent tobacco consumption and less severe dependence, whereas neglectful and indulgent styles are associated with more frequent consumption and greater dependence [17]; finally, a low socioeconomic status seems to increase the risk of adolescent smoking through its influence on parents' and peers' smoking behaviour [9].

Societal factors which might influence adolescents smoking include: restrictions on smoking, taxation, and costs [8, 9], for instance access to pocket money and ease of buying cigarettes increase the risk of adolescents smoking [18]; tobacco advertisement and media messages [8, 9], thus simply owning an item with a cigarette logo might increase the risk to smoke [18]; smoking behaviour of adolescents' role model [8, 9], like the misconceptions on the link between smoking and physically attractive appearance [18].

In this framework, we evaluated a sample of Italian adolescent students with the aim to verify which individual and social factors might influence their smoking status. Our focus was mainly addressed to significant adults and peers influence as well as knowledge and believes on smoking effects.

## 2. Methods

This was a school-based cross-sectional study conducted in 16 educational institutions located in 5 municipalities of Lombardy (North Italy). The study population included students of secondary and high schools. The school and students' response rate was 100 and 80%, respectively. Student response rate was calculated based on the number of students who participated in the survey, regardless of whether they answered all questions. Data were collected from 2001 to 2010. The study is part of an ongoing longitudinal project, coordinated by the Department of Prevention for Tobacco Dependence—prevention area—of Lombardy (North Italy). The project has the aim to verify the effects of a psychoeducation program addressed to improve adolescents' knowledge of tobacco dependence and smoking cessation strategies.

Each subject who accepted to participate to the study filled an anonymous self-administered 17-item questionnaire collecting socio-demographic data, information on smoking status (assessed by means of the question: “in the last week how many cigarettes did you smoke?”), information on individual and social factors that might influence their smoking status. Each item had a yes/no answer. Questionnaire development workshop was conducted to finalize the questionnaire (i.e., check the final draft verifying that irrelevant questions were not asked and obtain feedback of the respondents on the questionnaire).

The survey does not require institutional review board approval. Informed verbal consent from the school authority was obtained after explaining the purpose of the study. Schools and students were free to decide whether they wanted to participate, and schools required parental consent for student participation. All students in the selected classes, regardless of whether they used tobacco, were eligible to participate in the survey. They were asked to complete the questionnaire after explaining the purpose of the study and the instructions to fill in it. Considering the sensitivity of the issue, the school authority was requested not to be present in the class during filling of the questionnaire. Approximately 20 minutes, during a class-period, were provided to fill in the questionnaire. Students were assured that the information provided would remain confidential and were encouraged to be truthful in their responses. They were informed that their participation was voluntary. Any student absent on the day of the survey was excluded from the study.

## 3. Statistics

From the initial sample of 3,251 subjects we excluded 807 students since they did not provide information on their smoking history. The final sample counted 2,444 subjects. Subjects were attributed to two different groups according to their smoking status. Those who declared to have smoked regularly at least 1 cigarette per day for the previous week were defined as current smokers; all the others were considered current nonsmokers. 

A bivariate comparison of current smokers and current nonsmokers was performed using the *t*-test for independent samples for continuous variables and the chi-square test for dichotomous variables.

We used multivariate proportional odds models with the smoking index as the dependent variable. Independent variables tested were those obtained administering the questionnaire. Analyses were adjusted for age, gender, and education.

Significance levels were set at *P* < 0.05 (two-tailed). All statistical analyses were performed with the SPSS 18.0 statistical package and SAS 9.0 software.

## 4. Results

### 4.1. Demographic and Smoking Characteristics

Among the 2,444 students analyzed, 1,382 were boys (56.7%) and 1,056 girls (43.3%) with a mean age of 14.32 (±1.384) years. About 30% of the students were attending the middle school (*n* = 822; 33.6%) while 1,622 (66.4%) were at high school.

Concerning the smoking status, 607 (24.8%) adolescents declared to be current smokers while 1,837 (75.2%) declared not to smoke currently. Among nonsmokers, 1,026 (56.0%) were boys and 807 (44.0%) were girls, the mean age was 14.04 (±1.137) years. Among smokers, 356 (58.8%) were boys and 249 (41.2%) were girls, their mean age was 15.17 (±1.687) years and the mean age of smoking onset was 13.40 (±1.550) years. 

There was no difference concerning the gender between smokers and nonsmokers, while smokers were significantly older than nonsmokers (smokers: 15.17 ± 1.69 years versus nonsmokers: 14.04 ± 1.14 years; *t* = 18.54; *P* = 0.000).

### 4.2. Bivariate Analyses

In Table 1 we compared smokers and nonsmokers regarding the main sources of possible influences on their smoking status. Awareness on the fact that nicotine induces addiction and the opinion that tobacco use should be fought seem not to differentiate the two groups.

### 4.3. Multivariate Logistic Regression Analyses

In the present paragraph we report the odds ratios (ORs) and 95% Confidence Interval (95% CI) resulting from the multivariate logistic regression analyses measuring the risk of being current smokers versus nonsmokers. The analyses were adjusted for age, gender, and education.

The presence of smokers in the family, in particular the father and relatives different from parents, was a strong predictor of adolescents smoking. Interestingly, the knowledge of teachers who smoke did not influence the smoking status while seeing teachers who smoke reached the statistical significance. The risk of being current smokers was also predicted by the influence of friends and the feeling of inferiority (i.e., a group of representations and affects that reflect an individual's self-devaluation in relation to other) (Table 2).

Regarding harmfulness of nicotine, those who are aware of nicotine dangerous action to health seem less likely to be current smokers (Table 3). Similarly, those who have a correct knowledge on the number of cigarettes harmful to health or consider pipe/cigar as dangerous as cigarettes are at a lower risk to smoke (Table 3).

When the multivariate logistic regression analysis was run including in the model all the statistically significant variables listed above, the predictors of adolescent smoking were: not knowing that pipe and cigar are as harmful to health as cigarettes, not knowing that second-hand smoke is harmful for kids' growth, seeing teachers who smoke, having family members who smoke, being under the influence of friends or under the influence of the feeling of inferiority, not knowing that nicotine is harmful for the foetus (Table 4).

## 5. Discussion

The present findings show that the lack of knowledge of smoking and second-hand smoking negative effects to health, seeing teachers or having relatives who smoke, being influenced by friends and by the feeling of inferiority are strong predictors of youth smoking. In particular, the strongest predictor seems to be the lack of knowledge of smoking and second-hand smoking effects on health while the influence of significant others follows. 

Our data on the knowledge of health risk of smoking find support in the literature. Several studies have documented positive effects, for instance, of the truth campaign [19]. Moreover, earlier studies showed that among young people exposure to truth is associated with an increase in anti-tobacco attitudes and beliefs [20].

Relatively few studies have focused on the knowledge about, attitudes toward, and tolerance of second-hand smoking among college students and young individuals [21]. Even less studies have evaluated the relationship between such a knowledge and smoking. In the general population, never smokers seem to be more likely to acknowledge the health risks of second-hand smoking compared with smokers [22]. However, although smoking students are significantly more likely than nonsmokers to be exposed to second-hand smoke and not to perceive exposure to second-hand smoke harmful to health [23], the effects of the knowledge/perception that second-hand smoke is harmful to health on adolescents smoking has not been investigated to date. Thus, the present results could work as a spin off in this field of research.

Seeing teachers who smoke is a widely documented predictor of early use of tobacco [14, 15]. Interestingly, knowing teachers who smoke does not influence adolescent smoking status; once again, significant adults seem to exert substantial influence on adolescent behaviour through modelling with their own smoking behaviour [8]. Similarly, having relatives who smoke is a predictor of adolescents smoking, consistently with the literature [5, 8, 11–13, 17, 18]. We found that father, but not mother, smoking status has an effect. This result agrees with some authors [12, 17] but not with others [13]. However, it is noteworthy to note that not every study made the distinction between father and mother smoking status [18].

The influence of friends has been largely documented: having friends who smoke increases the likelihood of smoking while having friends who do not smoke reduces such a risk [5, 8, 11, 13]. Although it is not completely clear which variable between peers and significant adults has the strongest influence on adolescent smoking, according to the present findings significant adults have a relatively stronger effect than friends. However, the literature on this issue is extremely heterogeneous probably because of differences among countries [14], for instance, significant adults have a stronger role than peers in China [13] and in Pakistan [11]. It is the other way around. The possible role of cultural difference should be considered since it is already relevant for college student motives to quit [24].

The present study has some limitations. First, a cross-sectional research design was used and causality cannot be determined. Second, the data represent only students in public middle and high schools in Lombardy; national as well as international multi-sites, including public and private schools, data collection might produce more generalizable results. Third, the data were collected through a self-reported and anonymous questionnaire introducing the possibility of information bias; though this is believed to be minimal since the sample size is large and the response rates exceed 80%, which is generally a safeguard against biases inherent in self-report. In addition, some students in schools were not surveyed and some were absent the day of the survey (for reasons other than refusal to participate in the study) introducing the chance of some non-response bias. However, we believe that the sample is representative of the population as the majority of students attend secondary and high schools in Italy and the student response rate was about 80%. Finally, we could not attain biomedical validation of the current smoking status of the respondents altough and no measurements of phase delays in circadian rhythmicity were collected although addiction negatively affects rhythmicity (e.g., quality of wakefulness and sleep) which, in turn, seems related to the risk of developing addictive behavior [25, 26].

Despite the above limitations, the present research represents an important step ahead to identify the variables to be targeted in prevention programs. Such programs should be addressed to both significant adults and adolescents. In the first case, campaigns should awaken adults that adolescents tend to replicate their behaviour; thus, their smoking is not only a problem for their own health and a disease itself but has dramatic implications on the health of their children and pupils. In the second case, campaigns should awaken adolescents on the truth of smoking and second-hand smoking harmfulness to health. These latter programs should be addressed to adolescent smokers and nonsmokers as well as to students and non-students in order to mitigate the negative effects of peers influence on smoking. This would possibly change the vicious circle due to the influence of smoking peers into a virtual circle related to the influence of no smoking peers.

Our findings stress the importance of youth smoking research in order to understand the influences, beliefs, and knowledge about smoking among youth and the relationship between these factors and the smoking status. This will provide a starting point in the development of effective smoking prevention interventions specifically addressed to adolescents.

## Figures and Tables

**Table 1:** Smokers versus non-smokers. Chi-square test.

	Smokers		Nonsmokers		
	*N*	%	*N*	%	*χ* ^2^
Sources of influence of smoking status					

Smokers in the family					
No one	158	27.87	770	43.90	74.13∗
Father	103	18.17	319	18.19	
Mother	76	13.40	151	8.61	
Other relatives	30	5.29	137	7.81	
More than one relative	200	35.27	377	21.49	

Knowing teachers who smoke					
Yes	265	69.37	416	49.94	40.14∗
No	117	30.63	417	50.06	

Seeing teachers who smoke					
Yes	470	78.20	973	53.46	114.87∗
No	131	21.80	847	46.54	

Primary influence					
Friends					
Yes	532	95.68	1639	99.33	35.48∗
No	24	4.32	11	0.67	
Family					
Yes	45	7.99	133	7.92	0.03
No	518	92.01	1546	92.08	
Desire to grow up					
Yes	180	31.47	671	39.03	10.52∗
No	392	68.53	1048	60.97	
Feeling of inferiority					
Yes	57	10.18	316	18.90	22.92∗
No	503	89.82	1356	81.10	

Believes on smoking and secondhand smoke harmfulness					

Nicotine is harmful to health					
Yes	555	92.65	1476	99.19	69.93∗
No	44	7.35	12	0.81	

Nicotine is harmful to pregnancy					
Yes	577	95.69	1796	98.25	12.77∗
No	26	4.31	32	1.75	

Nicotine is harmful to the fetus					
Yes	549	93.37	1799	98.79	52.99∗
No	39	6.63	22	1.21	

Second hand smoke is harmful to health					
Yes	437	74.57	1413	95.73	203.58∗
No	149	25.43	63	4.27	

Second hand smoke is harmful to kids' growth					
Yes	477	88.99	1731	97.30	64.51∗
No	59	11.01	48	2.70	

Knowledge on nicotine/smoke damages					

Number of cigarettes/day harmful to health					
Correct answers	122	20.93	496	28.97	14.31∗
Wrong answers	461	79.07	1216	71.03	

Pipe/cigar are harmful to health like cigarettes					
Yes	213	36.72	1025	58.44	82.51∗
No	367	63.28	729	41.56	

Nicotine induces dependence					
No	11	1.95	37	2.16	1.79
Yes, psychical	376	66.67	1139	66.61	
Yes, physical	120	21.28	333	19.47	
Yes, psychophysical	57	10.11	201	11.75	

Need to fight tobacco use					
Yes	138	33.17	410	28.22	3.83
No	278	66.83	1043	71.78	

^*^ *P* < 0.05 (two-tailed).

**Table 2:** Sources of influence of subjects' smoking status. Multivariate logistic regression analysis adjusted for age, gender, and education.

	OR	95% CI	*P*
Relatives			

Age	1.911	1.747–2.091	<0.0001
Gender	0.924	0.748–1.142	0.4670
Education	0.592	0.444–0.788	0.0003
*Smokers in the family *	2.394	1.842–3.111	**<0.0001**
*Father's smoking *	1.579	1.164–2.141	**0.0033**
*Mother's smoking *	1.002	0.703–1.428	0.9896
*Other relatives smoking *	2.060	1.295–3.278	**0.0023**

Teachers			

Age	2.201	1.932–2.507	<0.0001
Gender	1.357	1.013–1.819	0.0410
Education	0.154	0.102–0.233	<0.0001
*Knowing teachers who smoke *	1.345	0.985–1.839	0.0626
*Seeing teachers who smoke *	3.519	2.310–5.361	**<0.0001**

Individual and personal source of influence			

Age	1.898	1.733–2.079	<0.0001
Gender	1.020	0.823–1.264	0.8572
Education	0.520	0.390–0.695	<0.0001
*Influence of friends *	0.183	0.086–0.389	**<0.0001**
*Influence of family *	1.040	0.674–1.606	0.8582
*Influence of desire to grow up *	0.789	0.621–1.004	0.0543
*Influence of feeling of inferiority *	0.490	0.347–0.693	**<0.0001**

**Table 3:** Beliefs on smoking and second-hand smoke harmfulness to health and knowledge about nicotine/smoke damages. Multivariate logistic regression analysis adjusted for age, gender, and education.

	OR	95% CI	*P*
Believes on smoking and secondhand smoke harmfulness			

Age	1.763	1.594–1.949	<0.0001
Gender	0.976	0.772–1.233	0.8377
Education	0.962	0.497–0.964	0.0296
*Nicotine is harmful to health *	0.212	0.091–0.492	0.0003
*Nicotine is harmful to pregnancy *	2.293	0.807–6.513	0.1192
*Nicotine is harmful to the fetus *	0.275	0.116–0.651	**0.0033**
*Secondhand smoke is harmful *	0.282	0.187–0.427	**<0.0001**
*Secondhand smoke is harmful to kids' growth *	0.551	0.306–0.993	**0.0475**

Knowledge on nicotine/smoke damages			

Age	1.810	1.614–2.030	<0.0001
Gender	0.826	0.637–1.071	0.1487
Education	0.891	0.627–1.267	0.5216
*Number of cigarette/day harmful to health *	2.012	1.482–2.731	**<0.0001**
*Pipe/cigar are harmful to health like cigarettes *	0.601	0.466–0.774	**<0.0001**
*Nicotine induces dependence *	0.724	0.328–1.597	0.4239
*Need to fight tobacco use *	0.788	0.601–1.033	0.0845

**Table 4:** Multivariate logistic regression analysis of all the statistically significant variables, adjusted for age, gender, and education.

	OR	95% CI	*P*
Age	1.640	1.461–1.840	<0.0001
Gender	1.117	0.848–1.471	0.4307
Education	0.656	0.445–0.968	0.0337
*Smokers in the family *	1.743	1.309–2.322	**0.0001**
*Seeing teachers who smoke *	1.830	1.343–2.495	**0.0001**
*Influence of friends *	0.234	0.097–0.567	**0.0013**
*Influence of feeling of inferiority *	0.570	0.379–0.858	**0.0071**
*Nicotine is harmful to the fetus *	0.278	0.106–0.726	**0.0089**
*Secondhand smoke is harmful *	0.663	0.340–1.291	0.2268
*Secondhand smoke is harmful to kids' growth *	0.342	0.214–0.548	**<0.0001**
*Number of cigarette/day harmful to health *	1.259	0.926–1.713	0.1418
*Pipe/cigar are harmful to health like cigarettes *	0.478	0.365–0.625	**<0.0001**
